# Analyzing the Gaps in Breast Cancer Diagnostics in Poland—A Retrospective Observational Study in the Data Donation Model

**DOI:** 10.3390/diagnostics15172127

**Published:** 2025-08-22

**Authors:** Wojciech Sierocki, Ligia Kornowska, Oliver Slapal, Agata Koska, Gabriela Sierocka, Alicja Dudek, Claudia Dompe, Michał Suchodolski, Przemysław Keczmer, Magdalena Roszak

**Affiliations:** 1 Department of Continuing Education, University of Oxford, Oxford OX1 2JA, UK; 2 Department of Pathophysiology, Poznan University of Medical Sciences, 60-806 Poznan, Poland; 3 Nuffield Department of Primary Care Health Sciences, Oxford OX2 6GG, UK; 4 Patient Centric Solutions Spółka Z Ograniczoną Odpowiedzialnością, 17-240 Kuzawa, Poland; 5 Faculty of Medicine, Medical University of Warsaw, 02-091 Warsaw, Poland; 6 Department of Computer Science and Engineering, University of Notre Dame, Notre Dame, IN 46556, USA; 7 Second Department of General Surgery, Jagiellonian University Medical College, 31-008 Kraków, Poland; 8 Department of Endocrinology CMKP, Bielanski Hospital in Warsaw, 01-809 Warsaw, Poland; 9 Department of Immunology, Poznan University of Medical Sciences, 60-806 Poznan, Poland; 10 Doctoral School, Poznan University of Medical Sciences, 60-812 Poznan, Poland; 11 Greater Poland Center of Digital Medicine, Poznan University of Medical Sciences, 60-806 Poznan, Poland; 12 Department of Rheumatology, Rehabilitation and Internal Medicine, Poznan University of Medical Sciences, 61-545 Poznan, Poland

**Keywords:** breast cancer, diagnosis, prevention, Poland

## Abstract

**Background:** Breast cancer is a major health concern in Poland, with significant incidence and mortality rates despite national screening programs. This retrospective study aimed to evaluate critical aspects of breast cancer management, focusing on waiting times, treatment coordination, cancer characteristics, diagnostic testing, and staging. **Methods:** We retrospectively analyzed 587 medical records of breast cancer patients (585 female, 2 male) collected between March 2023 and June 2024 through a data donation model. Data included tumor characteristics (histological type, grade, stage, biological subtype, receptor status, Ki-67), diagnostic and genetic tests, and timelines of key events in the diagnostic and therapeutic pathways. **Results:** Although referral to first oncology consult (18 days) and MDT referral/admission to treatment (10 days) met NFZ guidelines, diagnosis to surgery (94 days) and diagnosis to drug treatment (109 days) were significantly delayed. No records showed oncology coordinator assignment or educational material provision. Clinically, invasive carcinoma NST (77%) and early-stage (IA/IIA, 61%) were prevalent, with Luminal B (HER2-negative) being the most common biological subtype. BRCA1/2 testing was common, but Oncotype DX was not. For 314 HR+ HER2- patients, stage IA (44%) was most common, with no BRCA1/2 mutations found. **Conclusion:** Breast cancer care in the Łódź voivodeship falls short of national guidelines due to long waiting times and poor care coordination, a problem worsened by incomplete data. Improving record-keeping and speeding up diagnostic and treatment pathways are crucial for better breast cancer management in Poland. While patient data donation can help analyze real clinical pathways, data completeness, and consistency remain challenges.

## 1. Introduction

Malignant tumors represent a critical global health concern, with breast cancer being the most prevalent cancer among women worldwide [1]. In Poland, breast cancer accounted for over 24% of all female cancer diagnoses in 2021, emphasizing its significant national impact [2,3]. While risk factors like age, genetics, and lifestyle contribute, a substantial proportion of cases occur without identifiable risks [4,5]. Despite national mammography screening programs, participation rates remain suboptimal at 37%, leading to a concerning disparity between breast cancer incidence and mortality in Poland [6]. This deficiency hinders early detection, a critical factor in improving breast cancer outcomes, compounded by socioeconomic disparities that contribute to unequal screening access. The economic impact of cancer care in Poland is substantial, with expenditures surpassing PLN 15.5 billion in 2022 [7], underscoring the necessity for optimized oncology services.

To address these challenges, Poland has implemented the National Oncology Strategy (NSO) for 2020–2030 to enhance cancer prevention, diagnosis, and treatment through strategic investments and a patient-centric approach [8]. A key component of the NSO is the National Cancer Network (KSO), which seeks to standardize and improve the quality of oncology care across the country [9]. Despite initiatives such as the Diagnostic and Oncological Treatment (DiLO) card, which aims to expedite diagnosis [10], Poland still deals with delays and faces challenges in ensuring timely access to diagnostic tools [11]. Central to the patient’s treatment journey is the multidisciplinary team (MDT) of experts, which typically includes an oncologist, surgeon, radiotherapist, and nurse [12]. This team collaborates to establish a comprehensive treatment plan tailored to the individual patient’s needs. Following their decision, an oncology coordinator is assigned to supervise and facilitate the patient’s treatment process, ensuring continuity of care. This collaborative approach aims to deliver coordinated, patient-centered breast cancer management care. However, inconsistencies in medical record documentation suggest a deviation from these established procedures, potentially impacting the quality and coordination of patient care [13]. Moreover, despite national guidelines mandating specific timelines for diagnosis and treatment, delays are still present, highlighting a significant gap between policy and practice, with factors such as health center organization, staff availability, and patient location contributing to these disparities.

This study retrospectively analyzed 587 medical records of Polish breast cancer patients, with a specific focus on the hormone receptor-positive, HER2-negative (HR+ HER-) subtype, to assess critical aspects of care, including waiting times, treatment coordination, cancer characteristics, diagnostic testing, and staging. By examining these factors, we aim to provide a comprehensive overview of breast cancer management in Poland, identify areas for improvement, and contribute to the ongoing efforts to enhance patient care and outcomes.

## 2. Materials and Methods

### 2.1. Data Acquisition and Patient Recruitment

This retrospective observational study utilized data collected by the Donate Your Data Foundation (Fundacja Podaruj Dane) between March 2023 and June 2024. Data were acquired through a data donation model, where patients provided voluntary informed consent and power of attorney to access their medical records on their behalf. This model ensured compliance with GDPR and the highest ethical standards, allowing patients to retain control of their data. Patient response to the data donation campaign was overwhelmingly positive, with high enrolment and participation rates from breast cancer patients. Patients primarily asked about consent withdrawal, data security, and privacy, generally recognizing the value of sharing their medical history for scientific discovery and to help others. However, word choice was important. Some patients found “medical data donation” unsettling, associating it with “organ donation” or GDPR concerns. The Foundation adapted its language, using phrases like “share their medical history to help study the disease” to address hesitancy, clarify patient rights, and guide the informed consent process.

Medical records were obtained from patients treated at oncology centers within the Lodz voivodeship, specifically Salve Medica sp. z o.o., sp. k; Medica sp. z o.o., sp. k; District Health Center in Brzeziny Ltd.; M. Kopernik Regional Multispecialty Oncology and Traumatology Center in Lodz; Specialized Oncology Hospital in Tomaszow Mazowiecki; and Polish Mother’s Memorial Health Institute in Lodz. The M. Kopernik Regional Multispecialty Oncology and Traumatology Center and the Polish Mother’s Memorial Health Institute possess Breast Cancer Unit status. The care facilities were diverse in terms of size, urban vs. rural location, specialty level, and ownership structure (public and private with public contracts). Patient recruitment was conducted through diverse channels, including patient organizations, administrative staff (hospital registration, oncology coordinators), medical personnel (doctors, nurses), and online self-reporting. Inclusion criteria were confirmed diagnosis of breast cancer, C50, according to ICD-10, with the primary diagnosis, or recurrence of the primary cancer diagnosed, or metastasis of the primary cancer diagnosed after 1 January 2018, as well as informed consent.

The study included 587 breast cancer patient records (585 female and 2 male), with an average age of 61.7 years old, a range of 27–89 years, and a median of 64. Patients were classified as HR+, HER- if they had at least one receptor (ER—estrogen or PR—progesterone) with ≥10% expression. The breast cancer stage was determined using the TNM classification.

### 2.2. Data Management and Processing

The Foundation’s team retrieved medical records from treatment facilities through standard medical record-sharing channels, primarily in electronic format, with some paper records. To protect patient identity, all individual medical data were fully anonymized by the Donate Your Data Foundation before our access, with all personal and identifying information removed. As the data were fully anonymous and obtained from a third party where patients voluntarily donated their data, we received an official exemption (Sciences KB: 403/25) from bioethical approval from the Institutional Ethics Committee of Poznan University of Medical.

Our team applied to the Foundation for access to the anonymized data in accordance with their data access policy [14]. The Foundation’s Transparency Council, comprising representatives from two independent patient organizations, reviewed our application. Upon their approval, the Foundation granted us not-for-profit preferential access.

### 2.3. Data Structure and Variables

We employed the Aarhus checklist to guide our methodological approach to defining the intervals between key events in the patient diagnostic and therapeutic pathway [15] and to address potential biases and imprecisions in the medical records. Based on the analysis of the quality of the medical records obtained from facilities enrolled in the study and the nature of this study (retrospective medical records analysis), we decided to log the following events on the early diagnostic pathway:

- Suspected diagnosis, defined as the first time when a suspicion of breast cancer was raised in the medical records by a health professional.
- Initial oncology consultation, defined as the first consultation with an oncology specialist related to the current suspicion.
- Diagnostic confirmation, defined according to the recommendation of the European Network of Cancer Registries [16] as the date of the first histological or cytological confirmation of malignancy, which is the date when the pathology report was signed.
- MDT review, defined as the date of the MDT meeting. If the MDT review report was not included in the documentation, we assumed the date of the planned MDT review as the best approximation.
- First therapeutic intervention, defined as the first pharmacological or surgical therapeutic (not diagnostic) intervention related to the malignancy.

When medical records specified only the month and year of an event, the 15th of the month was assigned as the date. For events documented within a single quarter without a precise date, the midpoint of that quarter was used. For surgical procedures like mastectomy or breast-conserving therapy, where hospital records were unavailable, the procedure was assumed to occur on a single day. These derived dates were then used to calculate the duration between medical events. In other cases, when the medical records did not explicitly state the dates, we marked this information as missing—“data not available”.

Beyond the diagnostic pathway dates, we recorded patient demographic information and tumor characteristics such as histological type, TNM stage, NHG grade, biological subtype (determined based on immunophenotypic tests when not explicitly stated), ER/PR/HER2 receptor status, Ki-67 index, genetic testing (BRCA1/2, PIK3CA, Oncotype), and diagnostic tests (mammography, ultrasound, CT, MRI, etc.). Biological subtypes were determined based on immunohistochemical markers following the AJCC Cancer Staging Manual 8th Edition [17].

When the medical record indicated a referral for diagnostic tests or rehabilitation but lacked the test results or confirmation of the referral being carried out, we labeled such records as missing data. When evaluating the implementation of medical recommendations, it is crucial to recognize that a doctor’s referral does not guarantee the patient’s adherence. Factors such as the availability of medical services, financial constraints, lack of awareness regarding the necessity of the test, or the patient’s personal preference can influence the decision to undergo the recommended examination. Therefore, a comprehensive understanding necessitates monitoring the actual completion of diagnostic tests, not solely the issuance of referrals.

### 2.4. Statistical Analysis

Data used for inferential analysis have been processed and harmonized using Python 3.11 (Python Software Foundation, Wilmington, DE, USA). Cases lacking a complete AJCC 8th-edition TNM or stage were excluded from stage-based tests. Waiting-time interval (suspicion to first oncology visit) was calculated from cleaned date stamps; stage was dichotomized as early (0–IIA) versus advanced (≥IIB); histology was collapsed to five categories; and molecular subtype was assigned as Luminal A, Luminal B HER2-, Luminal B HER2^+^, HER2-enriched, or triple-negative. Ki-67 percentages were parsed numerically. Continuous, non-normal variables were compared with the Mann–Whitney U test (two groups) or Kruskal–Wallis with Dunn–Bonferroni post hoc (≥3 groups); categorical associations used the χ^2^ test with Cramér’s V; all the tests were two-sided with α = 0.05. The raw data contain 587 unique patients; however, each inferential test used analysis-specific list-wise deletion. A record was included only if all variables required for that particular model were present and internally consistent after data-cleaning.

## 3. Results

This study examined 587 medical records of breast cancer (C50) patients, focusing on waiting times, treatment coordination, cancer characteristics, and diagnostic testing, with a specific emphasis on the HR+ HER- subtype. It is important to note that all analyses were performed only on the data available within the medical records reviewed. To clarify, “data not available” in this study refers to the absence of documented information within the patient medical records reviewed. This does not imply that a specific test or procedure was not conducted, but rather that the medical records lacked the necessary documentation. Medical documentation was generally complete and robust, with the patient demographic information, tumor characteristics, and therapeutic interventions. Early diagnostic events and dates were often not recorded.

### 3.1. Waiting Times

**Figure 1:**
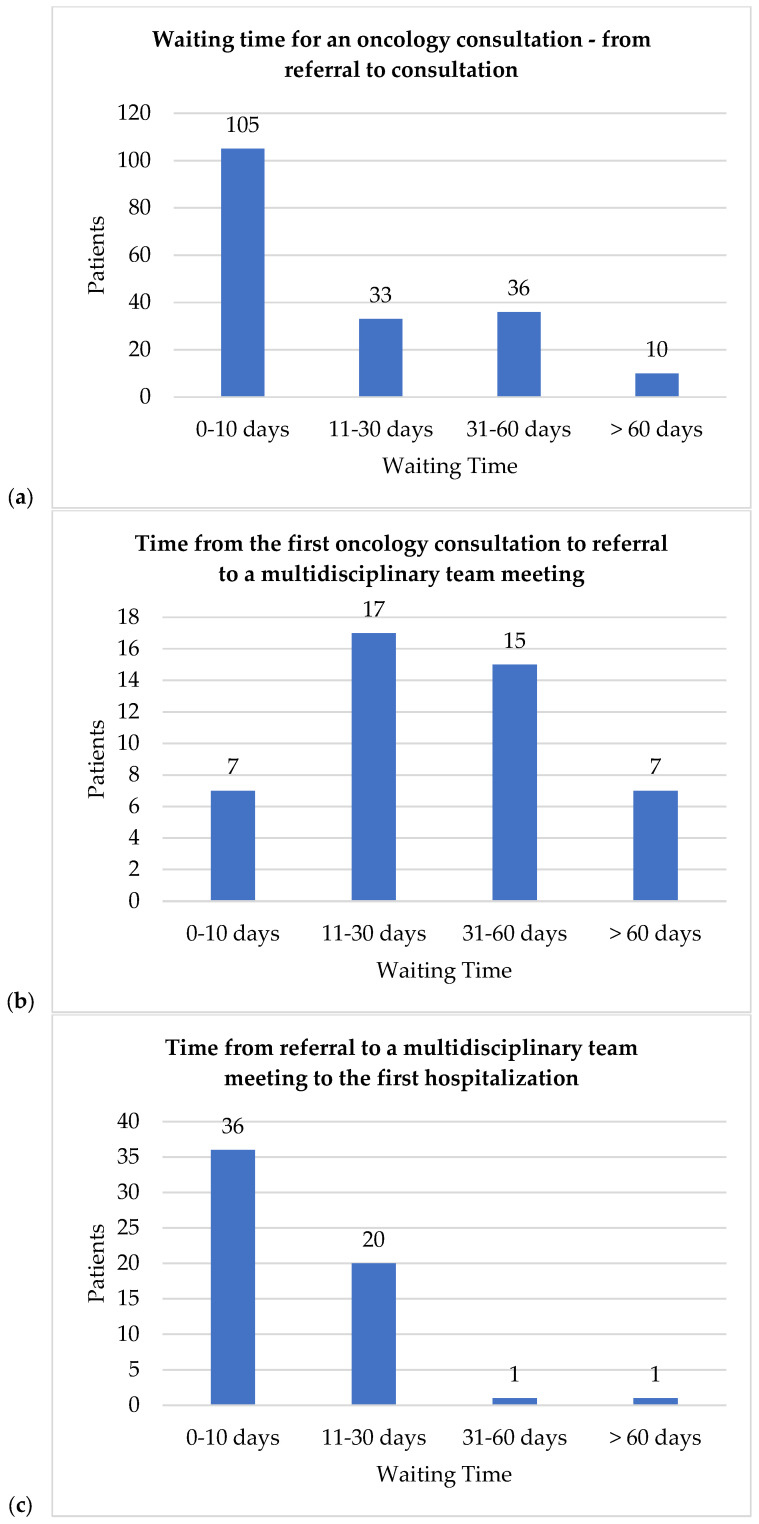

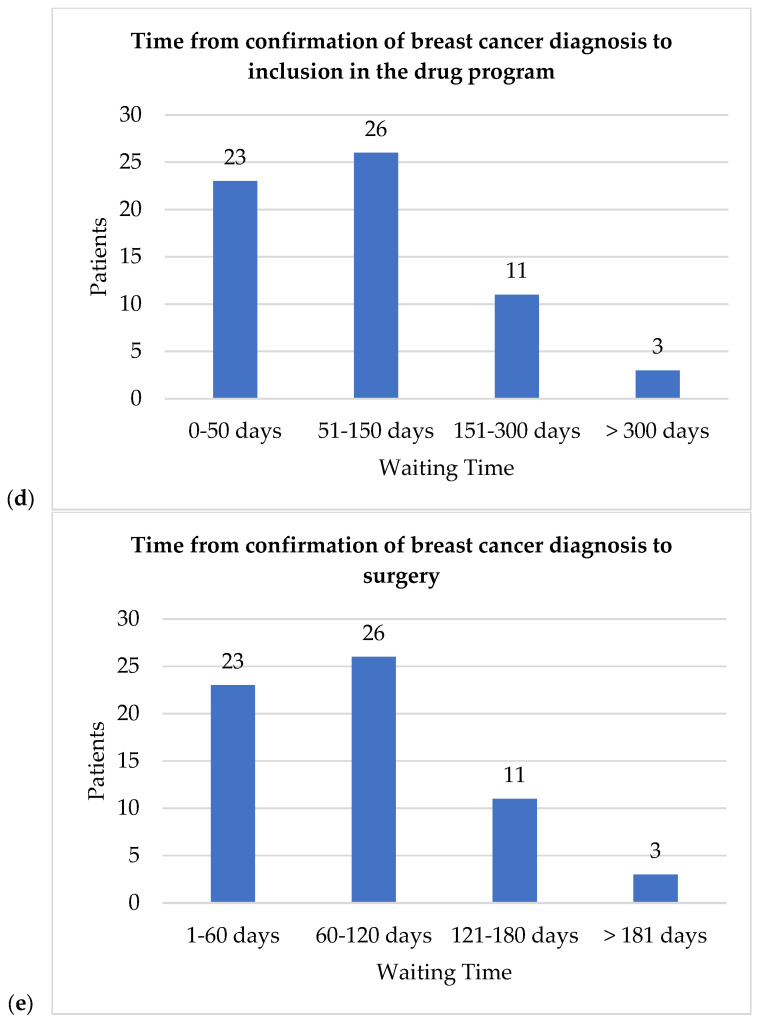
(**a**) Average waiting time for an oncology consultation (data available: 184; data not available: 403). (**b**) Time from first oncology consultation to referral to an MDT (data available: 46; data not available: 541). (**c**) Time from referral to an MDT to first hospitalization (data available: 58; data not available: 527). (**d**) Time from confirmation of C50 diagnosis to inclusion in the drug program (data available: 63; data not available: 522); (**e**) Time from confirmation of C50 diagnosis to surgery (550 patients underwent surgery). (Data available: 235; data not available: 315.)

We analyzed waiting times (Figure 1a), revealing that the average waiting time from referral to oncology consultation was 18 days, with a median of 6 days. Notably, 60 patients bypassed the referral process, presenting directly to an oncologist. The maximum waiting time was more than 3 months (97 days). The time from the first oncology consultation to the referral to an MDT meeting averaged 37 days (median 30 days) (Figure 1b). The time from the referral to the MDT meeting to the first hospitalization and therapy initiation averaged 10 days (median 7 days) (Figure 1c). Additionally, two patients were referred to the MDT after their first hospitalization. In both cases, the first intervention was surgery. The median time to inclusion in the drug program was 98 days (Figure 1d). Most patients (78%) began treatment within 150 days, and 37% within 50. Surgery occurred a median of 35 days post-diagnosis (mean 94, range 1–821; Figure 1e), with 45% of patients receiving it as their first treatment.

Among cases with complete timing data (*n* = 35), the median delay was 4 days (IQR 0–35) for early-stage disease (stage 0–IIA; *n* = 23) compared with 27 days (IQR 8–49) for advanced disease (≥IIB; *n* = 12). The difference did not reach statistical significance (Mann–Whitney U = 97, *p* = 0.153), and the effect size was small (*r* = 0.24). Table 1 summarizes key waiting times observed in our study, comparing them against existing NFZ and DiLO card system recommendations and assumptions for cancer care pathways in Poland.

According to declarations from the management, all 587 patients had been assigned oncological coordinators with higher education, and all patients received educational materials in paper form. However, a review of patient medical records did not confirm these declarations. No information about an assigned coordinator was found in any medical record, nor was there direct evidence of educational materials being provided.

### 3.2. Cancer Characteristics

The most common histological type of breast cancer was invasive carcinoma of no special type (NST) (77%), followed by invasive lobular carcinoma (ILC) (10%) and ductal carcinoma in situ (DCIS) (3%) (Table 2).

**Figure 2:**
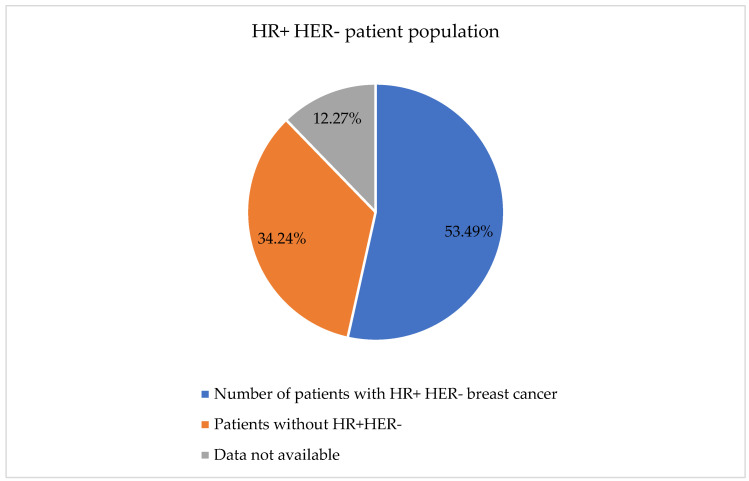
Demographics of the studied patient population (data available: 515; data not available: 72).

In total, 314 patients had the HR+ HER- subtype (Figure 2), with an average and median patient age of 60 years.

Histological malignancy grade, based on NHG classification, was predominantly G2—intermediate (66%), followed by G3—high status (20%) (Table 3). In the HR = HER population, histological malignancy was predominantly intermediate (68%), with 22% low and 10% high grade (Table 3).

Based on TNM staging, the majority of patients presented with stage IA or IIA disease (61%), with advanced carcinomas (defined as stage IIB/IIIA/IIIB/IIIC/IV) accounting for 36% of cases (Table 4). In the HR+HER- population stage IA was the most common in the HR+ HER- population (44%); 32.2% had advanced disease (IIB–IV), and 67.8% had low-grade tumors (IA–IIA) (Table 4).

**Figure 3:**
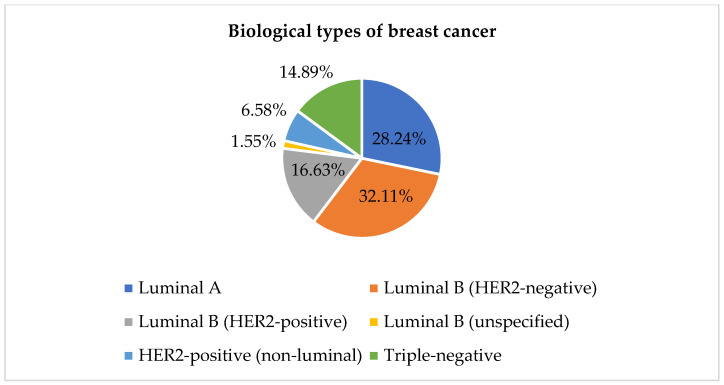
Biological types of breast cancer (data available: 515; data not available: 72).

The analysis of the biological subtype of breast cancer identified two patients with cancer of both breasts that differed in biological phenotype. As a result, the total number of biological subtypes was 517, with 515 patients studied. The results (Figure 3) identified Luminal B (HER2-negative) as the most prevalent subtype (166 cases), followed by Luminal A (146 cases).

A high percentage of analyzed medical records included tumor receptor testing data: ER (91%), PR (90%), HER2 (88%), and Ki67 (82%) (Table 5). In the HR+HER- population ER and PR tests were performed in 313 patients, and Ki67 in 292.

A five-level cross-tabulation of histology (NST, ILC, mucinous, apocrine, other) against stage group (early vs. advanced) showed no significant association (χ^2^ (4) = 2.34, *p* = 0.674). For example, NST accounted for 36/89 (40%) of advanced tumors versus 53/89 (60%) of early tumors.

When the cohort was stratified into Luminal A, Luminal B HER2-, Luminal B HER2^+^, HER2-enriched (non-luminal), and triple-negative breast cancer (TNBC), stage distribution differed significantly (χ^2^ (5) = 14.08, *p* = 0.015). Advanced disease was overrepresented in HER2-positive, non-luminal tumors (8/8, 100%) and in the small Luminal B subset lacking hormone-receptor expression (1/1, 100%), whereas only 38% of Luminal A cases presented at an advanced stage.

Ki-67 values (*n* = 121) varied markedly among subtypes (Kruskal–Wallis H = 33.6, *p* < 0.001). Median Ki-67 rose from 15% in Luminal A (*n* = 39) to 30% in Luminal B HER2- (*n* = 31), 50% in both Luminal B HER2^+^ (*n* = 21) and HER2-enriched tumors (*n* = 7), and peaked at 80% in TNBC (*n* = 23), confirming the expected biological gradient in proliferation.

### 3.3. Diagnostic Tests

Genetic testing was documented in both the general population and in the HR+HER- subgroup, including tests for BRCA1, BRCA2, CHEK2, PALB2, PIK3CA, NOD2, NBS1, and CDKN2A, with results presented in Table 6 and Table 7. In the HR+HER population, no BRCA1/2 mutations were detected. Oncotype DX testing was not mentioned in any of the reviewed records.

## 4. Discussion

This study analyzed 587 medical records of breast cancer patients in the Polish population from the Łódź voivodeship to assess waiting times, treatment coordination, cancer characteristics, diagnostic testing, and staging.

Despite national guidelines aiming for timely care, the study revealed significant challenges. Polish guidelines stipulate a maximum of 7 weeks from specialist consultation to diagnosis and 2 weeks from hospital presentation to treatment initiation [19,20]. Our study found the average time from referral to initial oncology consultation was 18 days, with 60 patients bypassing referrals, indicating variations in patient pathways. Furthermore, an average of 37 days passed between the first oncology consultation and the MDT meeting referral. While the time from the MDT referral to hospitalization and therapy initiation averaged 10 days, the average time to drug program inclusion and surgery was 109 and 94 days post-diagnosis, respectively. Our findings demonstrate that while initial intervals like referral to first oncology consultation and MDT referral/hospital admission to treatment start largely align with existing general guidelines, critical delays emerge later. The prolonged waits from diagnosis to surgery and drug treatment inclusion significantly exceed the spirit of timely care implied by current broad recommendations. This underscores Poland’s lack of comprehensive, clearly defined national standards for all key cancer care waiting times, particularly for specific treatment modalities following diagnosis. Our results strongly suggest an urgent need for the development and implementation of more granular, standardized national guidelines to ensure timely care and facilitate robust comparisons.

These delays suggest a gap between policy and practice, influenced by factors like health center organization, staff availability, and patient location. The DiLO card system, designed to expedite cancer care, may underperform, with reports showing a decline in initial cancer diagnostic timeliness in 2022 [21]. This has sparked debates between the Ministry of Health and the Nationwide Oncological Federation, exposing systemic issues. The KSO, aiming for full implementation in 2025, intends to address these issues via a tiered system of specialized oncology centers and e-DILO cards [22]. However, pilot data from the KSO reveal significant regional disparities in diagnostic and treatment timeliness. Findings from the Supreme Audit Office (NIK) audit (2019–2023) indicate that comprehensive oncology care and equitable access to publicly funded services were not achieved despite long-term programs like the National Programme for Combating Cancer (NPZChN) and the KSO [23]. The NIK highlighted failures in improving early cancer detection, standardizing pathology diagnoses, establishing quality metrics, and creating a new organizational structure. Further evidence of this gap is seen in the analysis presented in this article, where despite healthcare providers reporting universal oncology coordinator assignment and educational material provision, no information about an oncology coordinator appears in any of the medical records surveyed, possibly showing a poor correlation between reported practice and documented implementation.

In terms of cancer characteristics, invasive carcinoma NST was the predominant histological subtype, consistent with global and Polish data [24,25]. Similarly, lobular carcinoma and ductal carcinoma in situ also aligned with prior national data, marking them, respectively, as the second and third most frequent breast cancers [25]. Early-stage disease (as per TNM staging) was frequently observed, likely due to increased awareness and screening practices [26,27]. However, advanced-stage cases still comprised a significant proportion, underscoring the continuing need for improved strategies to address the challenges posed by advanced-stage breast cancer.

The prevalence of intermediate-grade (G2) tumors was consistent with typical breast cancer presentations [28]. Recognizing the heterogeneous nature of G2 tumors, characterized by a broader spectrum of phenotypes and prognoses compared to low- and high-grade counterparts [29], gene expression profiling tools like Oncotype Dx have been investigated to refine risk stratification [30,31]. While international guidelines strongly endorse the use of genomic assays like Oncotype DX for personalized treatment decisions [32], their full integration into the Polish healthcare system faces specific hurdles. Notably, despite recognition in national adaptations of guidelines [33], Oncotype DX has historically lacked consistent public reimbursement in Poland, leading to significant access limitations for patients [34]. Hence, the absence of Oncotype Dx testing in our data may be attributed to its higher cost and extended turnaround time relative to conventional pathological assessments [35]. Beyond financial barriers, broader systemic challenges such as a lingering institutional distrust, underfunding, and limited access to specialized care within the Polish healthcare infrastructure can impede the adoption of such advanced diagnostics [36]. Efforts by Polish professional societies to adapt international guidelines highlight the recognition of these tests’ value, yet also implicitly underscore the ongoing need to overcome the practical and systemic barriers to ensure equitable access and optimal patient care across the country.

Genetic testing data revealed frequent BRCA1/2 testing, underscoring its importance in assessing breast cancer risk. BRCA1 and BRCA2 mutations contribute to approximately 10–15% of all breast cancers. Women with a BRCA1 mutation face a significantly increased lifetime risk of breast cancer, with estimates ranging from 60% to 72%, while those with a BRCA2 mutation have a high lifetime risk of 55% to 77% [37]. Our analysis was conducted on an unselected breast cancer patient population, meaning recruitment did not involve pre-screening based on clinical criteria typically indicative of hereditary breast and ovarian cancer, such as strong family history, early-onset disease, or specific tumor subtypes (e.g., triple-negative breast cancer) [33].Consequently, the observed mutation prevalence reflects that of a general breast cancer population, rather than a high-risk, pre-selected cohort. This finding aligns with the significantly lower BRCA1/2 mutation rates typically reported in unselected populations compared to those identified through risk-based screening [38,39]. Our results highlight the impact of patient selection strategies on the observed prevalence of *BRCA1/2* pathogenic variants.

Despite National Comprehensive Cancer Network (NCCN) and NSO recommendations for genetic testing of CHEK2 and PALB2, in addition to BRCA1/2, for individuals with a >30% risk, the observed utilization and positive results for these and other cancer predisposition genes (P53, NOD2, NBS1, CDKN2A, PIK3CA) were unexpectedly low [8,33]. Research suggests that high- and moderate-risk gene mutations, like BRCA, CHEK2, and PALB2, are found in patients outside NCCN criteria, impacting prevention, treatment, and family risk [40,41]. This has led to recommendations from multiple studies, including the American Society of Breast Surgeons, for broader genetic testing, encompassing BRCA1/2, PALB2, and other appropriate genes based on the clinical picture, for all patients with a history of breast cancer [42]. While expanding genetic testing could identify more mutation carriers, it also raises concerns regarding cost-effectiveness and the potential for misinterpretation. Therefore, testing decisions should be personalized and guided by a thorough medical consultation.

This study also found a predominance of Luminal A and Luminal B (HER2-negative) breast cancer subtypes, emphasizing estrogen and progesterone receptor signaling. Both subtypes are characterized by estrogen receptor positivity, suggesting that hormone therapies targeting these pathways may be effective for many of these patients [32]. Consequently, accurate determination of ER and PR status is paramount for clinical decision-making [43]. The high ER and PR testing frequency in our results reflects the clinical relevance of hormone receptor status. This study’s high rate of Ki-67 testing underscores its clinical relevance as a proliferation marker in breast cancer management [44].

The HR+ HER- subtype represents the most common breast cancer subtype in women diagnosed with early-stage breast cancer, as confirmed by our results, where stage IA was the most common [45]. This subtype encompasses tumors with varying prognoses, further refined by factors like tumor grade and Ki-67 proliferation index, explaining the high frequency of its testing. The Ki-67 index is a well-established tool for risk stratification in breast cancer, differentiating between Luminal A and B subtypes and guiding treatment decisions [46]. However, while Ki-67 has been a focal point for decades, offering insights into outcomes and treatment responsiveness [47,48], the lack of consensus on optimal cut-off values and concerns about interlaboratory reproducibility and incremental prognostic value present challenges [49,50]. The low prevalence of BRCA1/2 mutations within the HR+/HER2- subtype may suggest that other genetic or molecular mechanisms drive tumor development in this population. BRCA1/2 mutations occur in 2–8% of HR+ breast cancers [51,52].

Our study utilized data gathered via a delegated access data donation mechanism. Data subjects gave their consent and a limited power of attorney to a trusted non-profit, allowing data to flow directly from data controllers to the delegate, without the data subject intermediation. This design aligns with the legal–technical architecture of the European Health Data Space (EHDS), which enshrines each EU citizen’s right to transmit their electronic health data to a chosen recipient or to empower a delegate to do so via state-operated proxy services [53]. Complementarily, the Data Governance Act formalizes “data-altruism” organizations, enabling them to aggregate such mandates for research in the public interest [54]. Poland is already preparing for this paradigm through a nationwide network of Regional Digital Medicine Centers (RDMCs) [55], established under a 2023 national standard to collect and pseudonymize routine hospital data and, once linked to the national IKP (Internetowe Konto Pacjenta) platform, whose mandate (Upoważnienie) workflow already allows citizens to grant third-party access to their health records, poised to adopt EHDS-compatible delegated access plumbing [56].

This study has several limitations. Its retrospective design and reliance on medical record data introduce biases and limit generalizability. We encountered significant incomplete data, particularly for initial diagnostic tests, confirmed referrals, and accurate early treatment dates. This “data not available” issue highlights a key limitation of data donation for mapping patient journeys, especially during early diagnosis. While donated data are valuable, its incompleteness can hinder comprehensive patient pathway assessment, risk stratification, and robust predictive modeling. This emphasizes the need for diverse data collection to fully understand complex patient movements. Furthermore, the study’s focus on Lodz province limits national applicability, exacerbated by an overrepresentation of less advanced cancer cases. This overrepresentation likely stems from selection bias inherent in an opt-in model, potentially skewing patient population representation.

Despite these limitations, the findings have several clinical and practical implications. Improving documentation practices, reducing waiting times, and enhancing treatment coordination are crucial for optimizing breast cancer care. Future research should focus on prospective studies with standardized data collection, investigating the impact of Oncotype DX testing on treatment outcomes and developing strategies to improve data completeness.

## 5. Conclusions

In conclusion, this study provides valuable insights into the management of breast cancer patients, including those with the HR+ HER- subtype. It also provides a disease-specific proof of concept that a delegated access data donation mechanism can deliver large, richly annotated routine health data sets for oncology research. While promising, this mechanism is vulnerable to operational frictions, including record heterogeneity, missing data points, incoherent coding, and selection bias due to an opt-in mechanism. These significant methodological limitations underscore the need for improved data structuring, collection, and documentation practices in further delegated access data donation projects under the EHDS framework. Addressing these gaps and implementing strategies to optimize care delivery are crucial for enhancing patient outcomes. Continuous quality improvement initiatives are essential for ensuring high-quality breast cancer care delivery.

## Figures and Tables

**Table 1:** Comparison of suggested time targets with observed waiting times.

Pathway	Recommendations	Observed Mean/Median (Days)	Comparison
Referral to First Oncology Consultation	Implicitly covered by overall diagnostic aims.	18/6	Efficient initial assessment.
Initial Oncological Diagnostics	NFZ: Max 28 days.	Not a direct equivalent in our data, but components like “Specialist consultation to diagnosis” contribute to this phase.	Our “Specialist consultation to diagnosis” (37 days) may exceed this.
Specialist Consultation to Diagnosis	DiLO card system: Max 7 weeks (49 days) [18].	37/30	Well within 49-day overall diagnostic assumption.
In-Depth Oncological Diagnostics	NFZ: Max 21 days (type, stage, metastases) [19].	Not a direct equivalent in our data, but components of “Specialist consultation to diagnosis” or subsequent diagnostic phases would apply.	Specific data not separately reported.
MDT Referral/Hospital Admission to Treatment Start	NFZ: Max 14 days from hospital arrival.	10/7	Within 14-day guideline; efficient initiation.
Diagnosis to Surgery	NFZ: Treatment start within 14 days of hospital admission/MDT decision.	94/35	Longer than general treatment start; highlights surgical challenges.
Diagnosis to Drug Treatment Inclusion	NFZ: Treatment start within 14 days of hospital admission/MDT decision.	109/98	Longer than general treatment start; reflects specific drug/patient factors.
Overall Time: Specialist Consultation Waiting List to Diagnosis	DiLO card system: Max 7 weeks (49 days).	Partially covered by “Specialist consultation to diagnosis” but full “waiting list” component not specified in your data.	Our “Spec. Consult. to Diagnosis” (37 days) aligns with this overall aim.

**Table 2:** Histological types of breast cancer (data available: 509; data not available: 78).

Histological Type of Breast Cancer	Number of Patients
No special type (NST)	391
Invasive lobular carcinoma (ILC)	49
Ductal carcinoma in situ (DCIS)	16
Mucinous carcinoma	6
Phyllodes tumor	3
Papillary carcinoma	3
Lobular carcinoma in situ	1
Tubular carcinoma	1
Cribriform carcinoma	1
Metaplastic cancer	1
Medullary carcinoma	1
Invasive cribriform and ductal carcinoma	1
Apocrine carcinoma	1
Apocrine adenocarcinoma	1
Angiosarcoma	1
Other	27

**Table 3:** Histological malignancy stage according to NHG in the general cancer patient group (data available: 442; data not available: 145 records) and in the HR+HER- subtype group (data available: 257; data not available: 57).

Histological Malignancy Stage According to NHG Classification	Number of Records
General breast cancer patients	
G1—low	64
G2—intermediate	290
G3—high	88
HR+, HER- subtype patients	
G1—low	55
G2—intermediate	176
G3—high	26

**Table 4:** The number of patients at the breast cancer stage in the general population (data available: 495; data not available: 92) and in the HR+HER- subtype group (data available: 292; data not available: 22).

Staging Determined by TNM Classification	Number of Patients
General breast cancer population	
Grade 0	8
Grade IA	176
IB degree	6
Grade IIA	127
Grade IIB	83
Grade IIIA	60
Grade IIIB	12
Grade IIIC	15
Grade IV	8
HR+HER- subtype patients	
Grade 0	0
Grade IA	129
IB degree	3
Grade IIA	66
Grade IIB	44
Grade IIIA	27
Grade IIIB	8
Grade IIIC	12
Grade IV	3

**Table 5:** Markers of predictive and prognostic factors in the general breast cancer patient group and in the HR+, HER- subtype patient group.

Markers of Predictive and Prognostic Factors		
	Test Performed	Data Not Available
General breast cancer patients		
ER	532	55
PR	529	58
HER2	519	68
Ki67	483	104
HR+, HER- subtype patients		
ER	313	1
PR	313	1
Ki67	292	21

**Table 6:** Genetic tests in the general breast cancer patient group and in the HR+, HER- subtype patient group: a positive test result (+) indicates the presence of a mutation, while a negative result (−) indicates the absence (data available: 176; data not available: 411).

Genetic Testing	Number of Patients
General breast cancer population	
BRCA1 (+)	9
BRCA1 (−)	167
BRCA2 (+)	1
BRCA2 (−)	171
CHEK2 (+)	6
CHEK2 (−)	136
PALB2 (+)	1
PALB2 (−)	137
P53 (+)	0
P53 (−)	1
NOD2 (+)	0
NOD2 (−)	1
NBS1 (+)	0
NBS1 (−)	1
CDKN2A (+)	0
CDKN2A (−)	1
PIK3CA (+)	0
PIK3CA (−)	0
HR+, HER- subtype patients	
BRCA1 (+)	1
BRCA1 (−)	88
BRCA2 (+)	1
BRCA2 (−)	87
CHEK2 (+)	3
CHEK2 (−)	70
PALB2 (+)	0
PALB2 (−)	69
P53 (+)	0
P53 (−)	0
NOD2 (+)	0
NOD2 (−)	0
NBS1 (+)	0
NBS1 (−)	0
CDKN2A (+)	0
CDKN2A (−)	0
PIK3CA (+)	0
PIK3CA (−)	0

**Table 7:** BRCA1 and BRCA2 genetic tests performed in the general breast cancer patient group and in the HR+, HER- subtype patient group.

Genetic Testing	Number of Patients
General breast cancer patients	
BRCA1 (+) BRCA2 (−)	3
BRCA1 (−) BRCA2 (−)	164
HR+, HER- subtype patients	
BRCA1 (−) BRCA2 (−)	87

## Data Availability

The data presented in this study are available on request from the corresponding author.

## Abbreviations

The following abbreviations are used in this manuscript:

NSO	National Oncology Strategy
KSO	National Cancer Network
HR+ HER-	Hormone receptor-positive, HER2-negative
ER	Estrogen Receptor
PR	Progesterone Receptor
TNM	Tumor, Node, Metastasis
NHG	Nottingham Histological Grade
NST	No special type
ILC	Invasive lobular carcinoma
DCIS	Ductal carcinoma in situ
TNBC	Triple-negative breast cancer
NIK	Supreme Audit Office
NPZChN	National Program for Combating Cancer
NCCN	National Comprehensive Cancer Network
EHDS	European Health Data Space
RDMCs	Regional Digital Medicine Centers
IKP	Internetowe Konto Pacjenta
